# Defying Food – How Distance Determines Monkeys’ Ability to Inhibit Reaching for Food

**DOI:** 10.3389/fpsyg.2016.00158

**Published:** 2016-02-18

**Authors:** Astrid F. Junghans, Elisabeth H. M. Sterck, Anne Overduin de Vries, Catharine Evers, Denise T. D. De Ridder

**Affiliations:** ^1^Selfregulation Lab – Department of Clinical and Health Psychology, Utrecht UniversityUtrecht, Netherlands; ^2^Animal Ecology, Utrecht UniversityUtrecht, Netherlands; ^3^Ethology Research, Biomedical Primate Research CenterRijswijk, Netherlands

**Keywords:** affordances, embodied cognition, inhibition, eating behavior, environmental influence, primates

## Abstract

Objects, such as food, in the environment automatically activate and facilitate affordances, the possibilities for motoric movements in interaction with the objects. Previous research has shown that affordance activation is contingent upon the distance of the object with only proximal objects activating potential movements. However, the effect of affordance-activating proximal objects on the ability to inhibit movements has been unaddressed. The current study addressed this question with two experiments on long-tailed macaques. In both experiments monkeys were situated behind a Plexiglass screen that prevented direct access to food placed right behind the screen. The food could only be reached via a detour through one of two holes on the sides of the screen. It was assessed whether monkeys’ ability to inhibit the unsuccessful immediate reaching movement forward toward the food depended on the distance at which the food was presented. Results of both Experiments revealed that monkeys reached for the proximally positioned food significantly more than for the distally positioned food, despite this Plexiglass screen preventing successful obtainment of the food. The findings reveal the effect of proximal, affordance-activating objects on the ability to resist movements involved in interacting with the objects. Implications for humans, living in environments in which proximal, or accessible food is constantly available are discussed. The findings can contribute to an understanding of why resisting accessible food in the environment is often unsuccessful.

## Introduction

A strong case has been made in the past decades for theories describing an organism’s functioning as the result of an interaction between mind, body, and environment (Wilson, 2002). Appropriate body-movements are not based on mere mental computations, but an organism’s functioning essentially depends on the environment surrounding it. Previous research involving a number of animal species and humans has shown that objects, including food, in the proximal environment automatically activate, and facilitate possibilities for motoric movements in interaction with the objects, so-called affordances (Costantini et al., 2011; Junghans et al., 2013).

While affordance effects have been shown consistently, it has recently led to the question of whether affordance activation upon exposure to tempting objects, such as food, may contribute to a reduced ability to resist reaching for them. If observing proximal food leads to automatic activations of reaching movements toward the food, it can be suggested that the movement may be more difficult to override by conscious, goal-driven processing than without automatic movement-activation. Considering that affordances are only activated by objects in the immediate, actionable environment, difficulties in consciously overriding movements should be observed only by proximal but not distant food (Cardellicchio et al., 2011). To test this idea, two experiments with long-tailed macaques investigated whether monkeys’ difficulties in inhibiting their reaching movements toward a presented food depends on the distance of the food. Monkeys represent a culturally and educationally unspoiled sample, whose response to food is unbiased by health concerns common to human samples. At the same time research has shown that monkeys and humans share neural responses related to reaching movements as well as action-selection mechanisms and action-inhibition processes (Sartori et al., 2014). This makes monkeys an ideal sample to investigate the effect of food in the environment on motoric responses only.

Affordances are possibilities for interaction, which activate the motoric system involved in an interaction between observer and object. These activations do not originate purely in someone’s mind, but in the environmental situation in which someone acts (Gibson, 1979; Wilson, 2002; Chemero, 2003). Affordances describe the effect in which the mere observation of an object facilitates an interaction by automatically preparing the motoric system for movements related to an observed object (Tucker and Ellis, 2001, 2004; Ridderinkhof et al., 2010). The positioning of a mug’s handle to the right side affords reaching movements with the spatially aligned right arm, which leads to an activation of the motoric system involved in such a movement (Bub and Masson, 2010).

Affordance perception in monkeys has been shown in both neurological and behavioral investigations. Previous research on Japanese monkeys has shown that activation patterns in movement-related neurons depend on how these objects are used, thereby revealing a neurological response to affordances of objects (Taira et al., 1990). Moreover, it has recently been discovered that neurons in the visuomotor area of the dorsomedial visual stream (V6A) in monkeys respond specifically to object affordances (Breveglieri et al., 2015).

On a behavioral level, monkeys have been shown to respond to affordances in objects by recognizing the opportunities these objects provide. Sartori et al. (2014) investigated the effect of different sizes of distractor objects on reaching-to-grasp movements and showed interference effects, such that observing distractor objects smaller or larger than the target affected grasping movements evoked by the target. This observation is compatible with the affordance theory. Gumert and Malaivijitnond (2013) revealed that long-tailed macaques, *Macaca fascicularis*, select stones on the basis of the most appropriate stone mass to process available food; thereby showing that monkeys perceive objects on the basis of actions they afford. Similar results have been obtained in gorillas, *Gorilla gorilla*, and orangutans, *Pongo pygmaeus* (Mulcahy et al., 2005), chimpanzees, *Pan troglodytes* (Hihara et al., 2006), as well as New Caledonian crows, *Corvus moneduloides* (Chappell and Kacelnik, 2004).

Similar to the findings in animals, affordance effects have been observed in humans based on more complex experimental designs. Studies based on stimulus–response compatibility designs have revealed shorter reaction times when a motor act is congruent with an observed object than when it is incongruent (Tucker and Ellis, 2001). For example, the presence of a mug with the handle to the right facilitates responses with the right hand rather than the left (Bub and Masson, 2010).

The FARS model (Fagg and Arbib, 1998) describes how affordances are computed in neurons in anterior intraparietal areas of the parietal cortex based on visual information derived from observing the mug. Resulting information about the required movements involved in interacting with the mug is passed on to an area referred to as F5, which is involved in grasping (Gentilucci et al., 1988; Arbib, 1997) and object observation. When an object is observed canonical neurons translate interaction-relevant information into potential motor actions regardless of the intention to execute the action or not (Jeannerod et al., 1995; Raos et al., 2006; Bonini et al., 2014). While these activations prepare for the (grasping) movements, organisms do not automatically respond to all affordances available to them in the environment. According to dual-process models action control processes combine automatic with more deliberative processes. In the above-mentioned example with humans the action-selection to grasp may be driven by the strong external stimulus of observing the mug. However, deliberative processing could interfere with these external effects and select action that is more appropriate or goal-relevant (Ridderinkhof et al., 2010). Generally, early stage processing depends more strongly on the automatic route and is thus more strongly driven by external stimuli, and therefore affordances, while later stage processing is more steered by deliberative processing, and thus reflective thought and self-control. This pattern is supported by the observation that fast responses to stimuli are more prone to error, because they lend themselves to the influence of task irrelevant features, compared to slow responses (Ridderinkhof et al., 2010). While the inhibition of activated motor movements has been reported to depend on processes in the subthalamic nucleus, it has also been shown the brain regions involved in the inhibition of activated movements depend on the elaborateness with which the action has been activated. Using a Go-No Go paradigm, recent research has shown that later stages of inhibition are accompanied by the activation of additional brain areas including the pre-supplementary motor area and the globus pallidus pars interna (Aron and Poldrack, 2006).

When it comes to food, these deliberative action-control processes may be affected by health considerations and dieting wishes; an influence unknown to monkeys. For that reason monkeys can be expected to show reaching movements to food that are unbiased by these deliberative considerations.

Studies based on human and non-human samples have shown that affordance effects depend on the spatial location of the object in reference to the observer. Objects need to fall into the peripersonal space, the area around the body that yields immediate interactions to activate canonical neurons that translate object features into action readiness (Costantini et al., 2010, 2011; Cardellicchio et al., 2011; Bonini et al., 2014).

In a previous study involving food, Junghans et al. (2013) showed that eating-related information was more strongly activated by the sight of proximal than distant food. Participants were shown images of proximal or distant food or other objects, followed by words relating to eating, observation, or other content. Participants’ task was to respond to words compatible with the observed picture. Thus, they were expected to respond to eating and observation words following food images. The results showed that participants were faster responding to eating words following proximal food than distant food. For observation words the distance of the food did not have an influence on response time. This indicated that eating-related information was more strongly activated by proximal than distant food.

These previous findings from research on both animals and humans consistently support the notion that proximal objects automatically activate a motoric readiness to interact with the object. The automatic nature of affordance activations suggests that movements activated by affordances should lead to difficulties in inhibition. If this assumption is correct, then reaching movements for proximal objects (which afford reaching movements) should be more difficult to inhibit than reaching movements to distant objects (which do not afford reaching movements). This hypothesis is tested in two experiments in monkeys.

If our assumptions are correct, our findings may extend previous research by showing that affordances activation is related to difficulties in inhibiting afforded movements. Moreover, findings may have important implications for strategies aimed at helping people to resist temptations, such as unhealthy food in the environment. In light of the current obesity epidemic many health promotions aim at supporting peoples’ self-control in resisting food in the environment; an attempt that may be hindered by affordance activation of proximal food.

## Study 1

### Methods

The first Experiment investigated the degree to which monkeys immediately reach for proximal and distant food presented behind a Plexiglas screen blocking access via the most direct, straight-forward reaching movement (Amici et al., 2008). The set-up of this task was designed in such way that it was necessary to inhibit the immediate forward reaching response in favor of a ‘detour’ through two holes on the left or right side of the Plexiglas screen to successfully obtain the food.

Monkeys were expected to show more immediate reaching movement straight toward the food when it was presented proximally than distally. In the proximal condition, the affordance effect of the food should automatically activate reaching. In this case action selection should be driven by immediate and automatic mechanisms resulting in reaching movements immediately forward to the food despite obstruction by the Plexiglas screen. In the distal condition, the food should not activate an affordance and therefore, the reaching movement should be more easily inhibited and a result of deliberative and intention-driven processes, which would allow the monkey to reflect on the situation and reach sideways through one of the holes to obtain the food (Ridderinkhof et al., 2010).

#### Participants

Sixteen healthy long-tailed macaques (five females; 11 males; the Haas-group) housed in a group of 25 animals at the Biomedical Primate Research Centre, The Netherlands participated in this study. The subjects’ age ranged from 2 to 20 years. They were all born in captivity. All monkeys were fed with monkey chow, fresh fruit, and vegetables, as well as bread and had constant access to water. The sawdust-covered cages provided enrichments in the form of fire hoses, ladders, tires, and pools. Monkeys had access to indoor and outdoor areas in their cages.

All subjects, but one, had participated in training and behavioral studies before and were familiar with clicker procedures, which means that they were familiar with the instruction technique. They were clicker-trained to move to the location where a trainer held a target (a plastic shoe-horn) against or through the fence of their cage: upon touching the shoe-horn, the trainer always made a clicker sound and a reward was given.

To alleviate suffering, the study took place in their home cage in which monkeys were individually tested in a corridor to which the experimental set-up was attached. None of the monkeys had previously participated in a study with similar design. They had access to food and water prior to and during the experiment (apart from 1 to 5 min during their trial in which only the experimental food was available). Furthermore, participation in this study was on a voluntary basis. Only those subjects voluntarily entering the area with the experimental setup participated in the study to ensure low stress levels. A maximum of two trials were conducted per monkey per day. Trials were terminated early and monkeys were returned to their group in the few cases in which monkeys showed signs of distress.

The study was approved by the Animal Ethical Committee of the BPRC (DEC755) and was carried out in accordance with the legal requirements of the Netherlands. All aspects of the studies were covered by this ethical approval.

No monkeys were sacrificed in relation to these studies. Upon termination of the experimental period, monkeys remained in their groups, and housing.

#### Material and Stimuli

**FIGURE 1:**
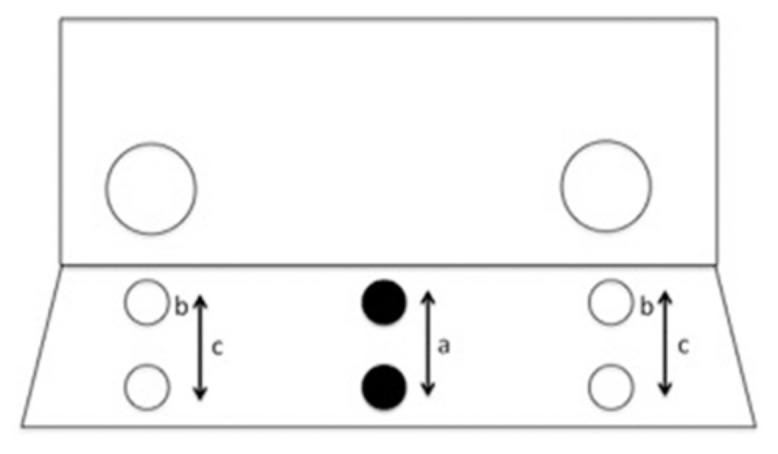
**Depiction of the position of food in experimental trials with a proximal or distal food position **(a)** as well as the position of food in training trials with fixed proximal position in Experiment 1 **(b)** and varied between proximal and distal in Experiment 2 **(c)****.

As previously employed by Amici et al. (2008), a Plexiglas screen was attached to the front of a separation compartment of the monkeys’ cages between the monkey and the experimenter. The Plexiglas screen had two 5.8 cm diameter wide holes on either side at a distance of 53.8 cm (See **Figure 1**). The size of the hole was sufficient for all monkeys to reach through comfortably. In front of the Plexiglas screen, on the experimenter side, a table was placed on which the food was presented. Prior to the experiment it was ensured that all monkeys in each group liked the target food (raisins).

#### Procedure

Each experimental session consisted of six practice training trials followed by one experimental trial. A maximum of two consecutive experimental sessions was run per day for each monkey. The limitation to two experimental trials per day was based on considerations regarding potential habituation and the desire to limit the trial duration to a minimum. In each experimental session monkeys voluntarily came to the corridor at the front of their cages where the experimental setup was placed and where they were separated from the group for a short period of time.

Training trials were carried out in order to teach the monkey about the physical properties of the Plexiglas screen and that they could reach the food through one of the holes. In each training trial the monkey was instructed with the shoe-horn to sit behind one of the holes and rewarded with a click when doing so, according to a semi-random order that was the same for each individual. Upon touching the shoe-horn, the monkey was presented with a raisin on a table placed right behind the hole in the Plexiglas screen (i.e., proximally). Once the monkey had reached for the raisin, the next trial started. In cases when monkeys did not reach through the hole spontaneously, the raisin was presented to them by holding it closer to the hole and occasionally presenting it through the hole. However, experimental trials were only conducted when the monkey had previously reached through the hole six times to obtain the food. For the experimental trial (trial 7) the monkey was clicker instructed to sit in the middle between the two holes. The raisin was then placed either proximally (10 cm behind the screen) or distally (25 cm behind the screen), in semi-random order, on the table in front of them (randomization was consistent across monkeys). The distal condition was chosen so that the food was difficult or impossible for the monkey to reach. If monkeys reached through one of the holes in the direction of the food but they had trouble grabbing it, the raisin was handed to them immediately.

Each volunteering monkey went through six experimental sessions of seven trials. Each session was video recorded for subsequent coding.

#### Dependent Variable

The dependent variable was the number of experimental trials the monkeys showed an onset of a reaching move directly forward toward the food, within 45° of a direct line between the monkey and the food, irrespective of the obstruction formed by the Plexiglas screen. The monkey could stop the movement before or was stopped when touching the screen. In addition, the movement had to occur within 2 s after exposure to or observation of the food. For the analysis the proportion of reaching movements out of all proximal and distal experimental trials were calculated.

To code the dependent variable two independent analysts coded the video footage of the experiments. Intercoder reliability was assessed with the second coder coding 25% of the data. For five experimental trials coders reached different conclusions. Those trials were subsequently conservatively coded opposite to the direction of the hypotheses.

### Results

**FIGURE 2:**
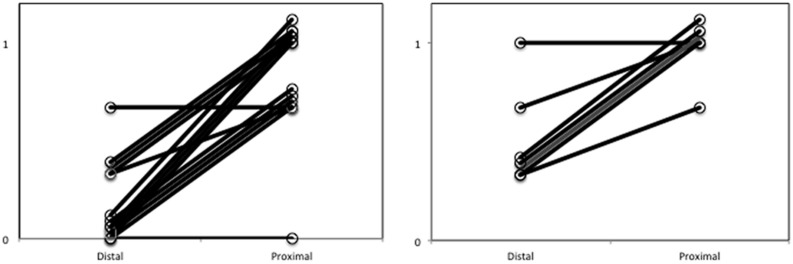
**Proportion of forward reaching movements out of all trials for each subject with proximally and distally presented food in Experiment 1 **(left)** and Experiment 2 **(right)**.** To enhance visibility of all lines representing subjects, small numerical additions have been made to scores on both the proximal and the distal value.

Paired samples *t*-tests were employed to examine whether the proportion of reaching movements in the proximal condition was higher compared to the proportion of reaching movements in the distal condition. As shown in **Figure 2**, results revealed a significantly larger proportion of reaching movements in the proximal (0.79) compared to the distal condition (0.14); *t*(14) = 7.24, *p* < 0.001; Cohen’s *d* = 2.57.

### Discussion Study 1

The analysis yielded support for the hypothesis that monkeys immediately reached for the food more often when it was presented proximally than distally despite the fact that they could not obtain the food using this movement.

Nevertheless, it could be argued that monkeys were more experienced in reaching for proximal food because the six practice trials presented food proximally rather than distally. This could have increased the likelihood for monkeys to reach for the proximal rather than the distant food and thus, presents an alternative explanation for the results. The second Experiment therefore added distant presentations of food to the first six practice trials to ensure that experience could not interfere with the results.

## Study 2

### Methods

#### Participants

To prevent training effects a new group of monkeys was tested for the second experiment. Housing situation, food availability, as well as familiarity with behavioral studies were similar to the previous group and treatments to alleviate suffering remained the same. Subjects included seven healthy long-tailed macaques (six females; one male; the Roza-group) housed in a group of 24 animals. The subjects’ age ranged from 4 to 11 years. As in Study 1, they were all born in captivity, had participated in training and behavioral studies before, and were familiar with shoe-horn instructions. Materials, stimuli, procedure, and dependent variable in the second experiment remained unchanged apart from including distal food to the six practice trials. Moreover, the food was always given to the monkey at the end of each experimental session irrespective of whether they had reached through the hole or not to ensure maintained motivation to participate. In half of the practice trials food was presented proximally, and in the other half it was presented distally, in randomized order. Intercoder reliability was assessed with the second coder coding 33% of the data reaching the same results. For one experimental trial neither could determine the correct code. For that reason it was conservatively coded opposite to the direction of the hypotheses. No monkeys were sacrificed.

### Results

Paired samples *t*-tests were employed to examine whether the proportion of reaching movements in the accessible condition was higher compared to the proportion of reaching movements in the inaccessible condition. As can be observed in **Figure 2**, results revealed a significantly larger proportion of reaching movements in the proximal (0.95) compared to the distant condition (0.48); *t*(7) = 4.26, *p* = 0.003: Cohen’s *d* = 2.19.

## General Discussion

In both experiments monkeys showed significantly more immediate reaching movements when the food was presented proximally than distally, thereby revealing the influence of proximal food on motoric activations. Despite the fact that this forward reaching for the proximal food could not have led to successful obtainment, monkeys did not successfully inhibit this movement in favor of the detour toward one of the holes in the Plexiglas or no movement altogether. Since previous research has shown that affordances only occur when an object is located proximally (within the peripersonal space) it can be reasoned that proximal objects facilitate automatic affordances and impede more successful, but indirect movement. It can thus be argued that the gravitational appeal that makes proximal, or accessible, food so difficult to resist, lies in the foods’ affordances, signifying the potential interactions the accessible food suggests to the observer.

The research contributes to the literature by showing differential effects of objects at different distances on motoric behavior in monkeys. While literature had shown affordance effects with monkeys on the basis of choosing appropriate tools (Cummins-Sebree and Fragaszy, 2001), effects of distance had been restricted to human samples (Cardellicchio et al., 2011).

Moreover, these findings appear particularly relevant when considering the implications they may have for human samples. The observation that in monkeys accessible food, located within one’s reach, leads to more uninhibited reaching movements than inaccessible food that is located just outside reach highlights the strong influence affordances can have on their failure to inhibit. In humans, a similar failure to resist proximal, or accessible, food in the environment has been observed and is often linked to an inability to successfully navigate the obesogenic environment (Allan et al., 2010). Affordances, exerted by accessible, but not inaccessible food, appear to be the most compelling mechanism underlying this effect. Affordances operate on an immediate, stimulus-driven level that often precedes deliberative processing including consideration of goals and self-control (Ridderinkhof et al., 2010). While people may have the aim to resist temptations, the motor activation of reaching for food occurs at an earlier motor stage that is less intention driven and may make resistance to temptations more difficult. As such, self-control processes, as they are commonly discussed in the literature on eating behavior (Muraven and Baumeister, 2000; De Ridder et al., 2011), are preceded by the activation of affordances, and may thus be less effective in overriding the already-activated motor plan of reaching for and eating observed food. This is not to imply that action control processes cannot prevent them, however, they need to be strong enough to override the immediate reaching impulse (Ridderinkhof et al., 2010).

The availability and accessibility of food in the obesogenic environment have a strong influence on what and how much people consume (Wansink, 2004, 2014). The current findings suggest that this effect could at least be partially explained by accessible food affording to be reached for on an automatic, motor level that makes deliberative processes such as self-control less successful in controlling food intake. The monkey results support this claim by showing that accessible food activates more immediate reaching movements and an inability to override these movements even though an obstacle will prevent its success.

Despite the clear and consistent findings, the Experiments were subject to a number of shortcomings. Firstly, while monkeys and humans share the same neurological mark-up when it comes to the activation of affordances and interacting with the environment (MacLean et al., 2014), the findings cannot directly be translated into results for humans. Obviously, humans are more capable than monkeys of resisting reaching toward food when an obstacle prevents successful reaching due to enhanced self-control capacities (MacLean et al., 2014). Nevertheless, the findings strongly suggest that affordances provide the mechanism by which motoric reaching for accessible food is activated and affordance effects have previously been found to influence both humans and monkeys (Tucker and Ellis, 2001; Sartori et al., 2014). Future research may involve conducting similar experimental designs with young children, who are less concerned with cultural considerations regarding food than adults and yet share their physiological and neural markup with older people.

Secondly, it could be argued that the initial reason for conducting the study on long-tailed macaques, the fact that they do not have health and dieting concerns, explains their inability to resist food. Obviously, the human food culture will play a role in better inhibition of motoric responses; however, this does not imply that the motoric activation has not taken place. The human ability to resist food better than monkeys should be based on healthiness considerations and dieting intentions, which should modulate the immediate and automatic reaching responses via online and anticipatory action control processes. Moreover, the activations may be influenced by an awareness in humans that food is no short resource. Thus, these considerations should provide the basis for enhancing or preempting activated movements (Ridderinkhof et al., 2010).

Third one could consider the plexiglas screen presented between the monkeys and the food an obstacle that hinders interaction. Previous research has indicated that affordance activation by objects depends indeed on the objects’ location within the peripersonal space; however, this peripersonal space was found to be determined by operational possibility to interact rather than a mere metric ability to reach the object (Bonini et al., 2014). They observed weakened affordance activation when objects placed within the peripersonal space were shielded by a plexiglas screen. However, considering that both our conditions were shielded by a plexiglas screen the differential effects for proximal and distal condition hold irrespective of these weakened affordances.

Finally, it could be argued that the findings of this study can be explained by competition between two alternative motor plans rather than by the inhibition of one motor plan. The observed affordance effect could thus be explained by two different mechanisms, the inability to inhibit the afforded movement and/or the larger impact of the forward reaching motor plan in contrast to the sideways reaching motor plan. These potential mechanisms underlying the affordance effect should be investigated in future research to holistically understand the drivers of affordance effects.

## Conclusion

The study shows that the observation of accessible food leads to less inhibition of reaching movements to obtain the food than the observation of inaccessible food in long-tailed macaques. This suggests an association between the affordances exerted by accessible food and a reduced ability to inhibit an activated movement. These findings may have explanatory implications for humans living in an environment with constantly accessible food. Despite the fact that people have the capacity to override motoric activations, the constant accessibility of food requires similarly constant action control. In light of the abundance of accessible food it is not surprising that peoples’ self-control fails eventually leading to increased consumption and weight gain.

## Author Contributions

The studies were designed by AJ, ES, AO, CE, and DR. Materials and procedures were determined by AJ, AO, and ES. Data collection was performed by AJ and AO. Data were coded by AJ and AO and analyzed by AJ. AJ, ES, AO, CE, and DR interpreted the results. The manuscript was drafted by AJ and revised by AO, ES, CE, and DR. AJ, AP, ES, CE, and DR approved the final version.

## Conflict of Interest Statement

The authors declare that the research was conducted in the absence of any commercial or financial relationships that could be construed as a potential conflict of interest.
